# Making the World Behave: A New Embodied Account on Mobile Paradigm

**DOI:** 10.3389/fnsys.2021.643526

**Published:** 2021-03-01

**Authors:** Umay Sen, Gustaf Gredebäck

**Affiliations:** Department of Psychology, Uppsala University, Uppsala, Sweden

**Keywords:** mobile paradigm, sensorimotor contingency, embodiment, infant memory, learning

## Abstract

In this review article, we describe the mobile paradigm, a method used for more than 50 years to assess how infants learn and remember sensorimotor contingencies. The literature on the mobile paradigm demonstrates that infants below 6 months of age can remember the learning environment weeks after when reminded periodically and integrate temporally distributed information across modalities. The latter ability is only possible if events occur within a temporal window of a few days, and the width of this required window changes as a function of age. A major critique of these conclusions is that the majority of this literature has neglected the embodied experience, such that motor behavior was considered an equivalent developmental substitute for verbal behavior. Over recent years, simulation and empirical work have highlighted the sensorimotor aspect and opened up a discussion for possible learning mechanisms and variability in motor preferences of young infants. In line with this recent direction, we present a new embodied account on the mobile paradigm which argues that learning sensorimotor contingencies is a core feature of development forming the basis for active exploration of the world and body. In addition to better explaining recent findings, this new framework aims to replace the dis-embodied approach to the mobile paradigm with a new understanding that focuses on variance and representations grounded in sensorimotor experience. Finally, we discuss a potential role for the dorsal stream which might be responsible for guiding action according to visual information, while infants learn sensorimotor contingencies in the mobile paradigm.

## Introduction

Throughout the first half of the 20th century, young infants were viewed as unformed versions of adults whose learning capacities were limited due, in part, to underdeveloped prefrontal structures (Hodel, 2018) and a lack of language (McGraw, 1932; Twitchell, 1965). Rovee-Collier was one of the pioneers against the idea that young infants are not able to learn (Branson, 2014). One day, while trying to stop her son from crying to study for her dissertation exams, she made a profound observation, one that would define her academic career. Her 1.5-month-old son had a mobile that she always used to distract him. On that day, she remembered her grandmother’s saying, “Oh, darling, if you could only harness the energy of a 2 or 3-year-old to run the windmills in Holland” (Rovee-Collier, 2006, p. 8), and thought that she could test this claim. She tied the silk belt on her dress to the mobile and the infant’s foot, so that whenever her infant moved his leg, he would activate the mobile. She observed that her son increased his kicking rate when his leg was tied to the mobile and stopped kicking when it was not. The observation of her son’s behavior led her to test the idea that young infants could learn new behavior by adjusting their actions to gain a reward. The results were remarkable: 2-month-old babies doubled, and in some cases tripled, their response rate in the first couple of minutes of the acquisition phase (Rovee and Rovee, 1969). However, they were also controversial. Reviewers noted that the topic was not interesting, as infant motor actions were recognized as “something a cockroach could learn” (reviewer response as reported by Rovee-Collier, 2006, p. 16) and at odds with contemporary theories (“these are wonderful data, but we do not believe them, because Piaget said babies can not do this”; Rovee-Collier, 2006, p. 9). Despite these arguments, the first article was eventually published (Rovee and Rovee, 1969), followed by more than 100 others using her invention, the mobile paradigm.

For more than 50 years, the mobile paradigm was used to study a wide range of topics, from perceptual abilities to the long-term memory capacity of young infants, challenging contemporary views on the capabilities of and learning opportunities available to young infants. Although a recent article (Jacquey et al., 2020a) reviewed the literature on contingency learning paradigms, including the mobile paradigm, to examine different determinants affecting learning sensorimotor contingencies, no review has yet attempted to integrate the entire range of studies using the mobile paradigm from Rovee and Rovee (1969) to today (Jacquey et al., 2020b; Zaadnoordijk et al., 2020). Additionally, the present review discusses a role for a new account on the mobile paradigm by offering a critical perspective on some aspects of this literature. In this article, we aim to review studies that have relied on the mobile paradigm to assess learning, motor development, memory, and cognition in early infancy, starting with a description of the core mobile paradigm itself and ending with presenting a different interpretation of the paradigm, one that focuses more on the embodied experiences of the infant and her movements.

## The Mobile Paradigm

The mobile paradigm is an operant conditioning procedure implemented by Rovee-Collier so that she could study infant memory development (for a detailed review of methods used to investigate infant memory see Rovee-Collier and Hayne, 1987; Hayne, 2004). In this procedure, the rate of stimulus presentation in response to the behavior is determined by a conjugate reinforcement schedule in which the reward is proportional to the amount of behavior exhibited (Lindsley, 1957; Rovee and Rovee, 1969). In Rovee-Collier’s (1996) terms, it allows the infant to shop for the value of the reinforcing stimulation they most prefer.

In practice, the procedure is as follows. After the infant is placed in a crib, a ribbon is attached to one of their legs. Two adjacent stands are mounted on the crib, one connected to a mobile, and the other is empty. The original (and most used) paradigm has three phases: baseline (3 min), acquisition (9 min), and extinction (3 min; Fagen et al., 1976; Sullivan et al., 1979). During the baseline and extinction phases, the infants are allowed to move their legs normally with their legs attached to the empty stand. During the acquisition phase, the leg is connected to the mobile stand and their movements set the mobile in motion. Rovee-Collier argued that, after operant learning took place (e.g., increased kicking rate in the first minutes of acquisition phase) by gaining control over the mobile, the environment continued to reward the infant, resulting in individual differences in movement with respect to how much the infant experimented with their surroundings. Not only the sensory consequences (e.g., haptic feedback in the leg, visual stimulation coming from the moving mobile), but “making the world behave” (Skinner, 1953, as cited in Rovee-Collier and Gekoski, 1979), in other words gaining control over the mobile, strengthened the stimulus-response associations.

## Breaking The Grounds

These ideas contradicted the prominent theories of the time. For example, Piaget (1952) observed his 2-month-old son Laurent while he was moving his arms connected to the toys hanging above his crib and interpreted these actions as expressions of joy, not as conscious coordination. Furthermore, Piaget (1952) thought that infants at this age can not be operantly conditioned (as cited in Rovee-Collier and Barr, 2001). For Rovee-Collier and Gekoski (1979), however, a similar type of behavior in the mobile paradigm indicated voluntary actions learned through reinforcement rather than increased excitement. The evidence supporting their argument demonstrates that the increased movement was specific to the limb connected to the mobile and did not occur in other limbs, contrary to the joy-based interpretation (Rovee-Collier et al., 1978). Also, when the ribbon was initially connected to one leg and then switched to the other, the learning pattern reversed. In other words, infants increased the kicking rate of the currently attached leg and decreased the kicking of the previously reinforced leg. These results revealed two important conclusions: neither proprioceptive feedback nor a joy reaction could explain the increase in kicking in the leg connected to the mobile, and young infants could adapt to their changing environment quickly and adaptively. Rovee-Collier (1996) interpreted this as an indication that infants prefer cost-effective actions to minimize their energy consumption.

## Extending The Paradigm

After the original mobile paradigm showed that young infants can learn a new motor behavior through operant conditioning (Rovee and Rovee, 1969; Rovee-Collier et al., 1978), the extensions of the original paradigm were developed for investigating learning and memory early in infancy. For instance, Watson (1979) conducted series of experiments where a pillow under the infant’s legs triggered the movement of the mobile. It was shown that the learning rate was differentially affected depending on the changing probability of non-contingent as well as contingent stimulation. Other extensions of the mobile paradigm were used for studying infant memory: a test of simple forgetting and reminder procedures (for a detailed review see Rovee-Collier and Hayne, 1987; Rovee-Collier and Barr, 2001).

**Figure 1:**
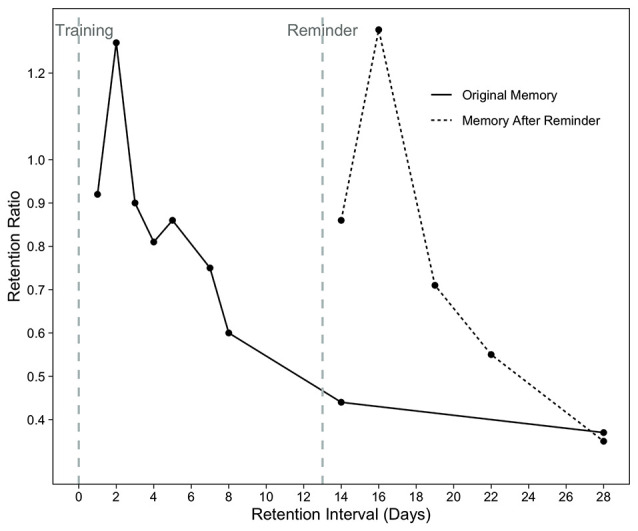
Retention ratios after 2 days of training (solid line) or 2 days of training plus a reactivation reminder (dashed line). Redrawn from Rovee-Collier and Sullivan (1980). Copyright (1980) The American Association for the Advancement of Science.

Test of simple forgetting measures retention, or how long information can be maintained in memory, which has been shown to range from a day to a week (Rovee and Fagen, 1976; Sullivan et al., 1979). In this test, memory is measured by reintroducing the infants to the training mobile after a retention interval. Two measures are used to estimate the amount of retention: baseline ratio and retention ratio. The baseline ratio indicates whether the response rate in the long-term retention test exceeds the baseline movement level. The retention ratio describes how much the infant’s response rate in the long-term retention test differs from the response rate in the immediate retention test that occurs immediately after the learning is complete. Forgetting rates of infants following the learning is determined by measuring retention ratios in different groups at varying retention intervals. This group-level analysis enabled the mapping of the timeline of forgetting in young infants (Figure 1).

There are two types of reminder paradigms, referred to as reactivation and reinstatement. The reactivation paradigm measures retention after longer intervals of up to several weeks by introducing a reminder after the memory is forgotten (i.e., when it becomes inaccessible). During reactivation, infants are reminded by the cues from the training episode by passively viewing a moving mobile between the training and test sessions to make a dormant memory accessible again (Rovee-Collier and Hayne, 1987). In the reinstatement paradigm, infants can move the mobile in the reminder session that occurs again between the training and test sessions (Borovsky and Rovee-Collier, 1990; Galluccio and Rovee-Collier, 1999; Adler et al., 2000). Though infants passively observe the mobile in the reactivation paradigm, reinstatement allows them to actively participate in the reminding.

## Memory

In the mid-20th century, the field of psychology went through a major transition period, but infant memory was not an often-studied phenomenon. Until 1965, the word memory was not even mentioned in Child Development abstracts (Kail and Hagen, 1977, as cited in Miller, 2014). At the time, behaviorism was the prevailing theoretical account for studying learning and memory, but the cognitive revolution was starting to change the field (Miller, 2003). Behaviorist accounts argued that the building blocks of learning and memory are stimulus-response associations. However, more cognitivist ideas of memory in general, and memory development, in particular, were emerging. For example, Tulving’s differentiation between semantic and episodic memory (Tulving, 1972) was one of the ground breaking ideas of the time that reconceptualized memory formation as more nuanced and complex than what was expected from reinforcement accounts. Similarly, new ideas about memory, such as context effects (Tulving, 1972; Tulving and Thomson, 1973; Godden and Baddeley, 1975), and the susceptibility of memories to change when encountering new information (Loftus, 1975, 1979) were emerging. Inspired by these ideas and taking an active role in the transformation of psychological science, Rovee-Collier carried out systematic investigations of these phenomena in early infancy, but her early work was still firmly grounded in behaviorism.

In their seminal article, Rovee and Rovee (1969) claimed that young infants can be operantly conditioned, as 3-month-old infants tripled the kicking rate within the first couple of minutes of the acquisition phase when the mobile was connected to their leg, assuming that the movement of the mobile reinforced the infants’ kicking. After successive days of training with the same mobile, infants remembered the same mobile 1 week following the training (Sullivan et al., 1979) and discriminated against a novel mobile (Fagen et al., 1976; Rovee and Fagen, 1976). These findings were the first to provide evidence that young infants can remember learned information after intervals far longer than previously assumed, meaning that young infants could store learned information for days, not only a couple of hours (Fagan, 1970, 1971). The retention capacity of young infants was found to increase with age, from 1 week at 2 months old (Vander Linde et al., 1985) to 13 weeks at 18 months old (Hartshorn et al., 1998a; Rovee-Collier and Hartshorn, 1999).

Using the reactivation paradigm, Rovee-Collier and her colleagues demonstrated that 2–3-month-olds can retain learned information for at least a couple of weeks when they were reminded between training and test sessions (Rovee-Collier et al., 1980; Fagen and Rovee-Collier, 1983; for a review see Rovee-Collier and Hayne, 1987). Observing the non-contingently moving mobile as a reminder (reactivation paradigm) increased the probability of remembering the training mobile to a degree that it would be remembered at the end of the training episode when the memory was just formed (Rovee-Collier et al., 1980, Figure 1). This suggests that, not only forgetting can be recovered due to a reminder, but also that the reminder leads to retention of memory as complete as just after training. Forgotten memories were recovered more quickly as infants got older, 24-times faster at 6 months of age compared to 3 months of age (Fagen and Rovee-Collier, 1983; Boller et al., 1990; for a review see Rovee-Collier and Hartshorn, 1999). When the infants were reminded periodically by activating the mobile themselves (reinstatement paradigm), both 3- and 6-month-olds remembered the training memory for 5 months (Rovee-Collier et al., 1999) and 18 months, respectively (Hartshorn and Rovee-Collier, 1997, as cited in Rovee-Collier and Hartshorn, 1999).

Together, these studies revealed the remarkable memory capacity in early infancy, which is taken for granted in the current understanding of development. Furthermore, they have a clear theoretical implication because they support the argument for a distinction between availability and accessibility in memory of young infants, which was initially discussed as a characteristic of adult memory (Tulving and Pearlstone, 1966; Tulving, 1972). According to this distinction, a failure to remember learned information may indicate that the trace of the memory is lost and the memory is not available anymore, or a failure to access the information stored in the memory. The forgetting of young infants was considered an accessibility failure rather than encoding or storage deficit because of their capacity to remember what they learned when they were reminded (Rovee-Collier and Hayne, 1987). Similarly, the memory performance of 2-month-old infants was not different from their older counterparts when they were given the opportunity to encode more cues during encoding (Vander Linde et al., 1985; Hayne et al., 1986). Therefore, even 2-month-olds could overcome the quick decay by sampling more cues during encoding, which is in sharp contrast to the idea that young infants are unable to retrieve stored information due to the immaturity of the central nervous system (Campbell and Spear, 1972) or long-term memory that has not been fully formed (Kagan and Hamburg, 1981, as cited in Schacter and Moscovitch, 1984; Olson and Strauss, 1984; Schacter and Moscovitch, 1984).

## Two Distinct Memory Systems

In the 1980s, research on adult memory pointed to dissociations between two memory types depending on whether the recollection of memory is a conscious process, mostly referred to as implicit and explicit (Graf and Schacter, 1985), or declarative and non-declarative (Cohen and Squire, 1980). The evidence for such a dissociation derived from amnesic patients’ impaired performance on explicit, but not implicit, memory tasks, and the two memory systems’ differential susceptibility to previous experience or priming (for a review see Schacter et al., 1993). Schacter and Moscovitch (1984) argued that such differentiation was not evident in the first 6 months of life, and memory that requires conscious recollection (also referred to as late maturing memory system or explicit memory) develops towards the end of the first year. On the other hand, Rovee-Collier (1997) argued that the test of simple forgetting and the reactivation paradigm measure two distinct memory systems that are fully functional even in the first months of life. An important example of such memory dissociation in young infants is the development of explicit memory with age, as measured with the simple forgetting test, whereas implicit memory capacity (e.g., reactivation paradigm) remains the same throughout development (Hartshorn and Rovee-Collier, 1997; Rovee-Collier, 1997).

The studies discussed so far provide evidence that, even at 2 months of age, infants have a remarkable capacity for learning sensorimotor contingencies and retaining what they have learned for days, and in some cases even weeks when they are reminded using cues from the training episode. Maturation has a role in memory development, as the retention capacity increases with age, but experience also impacts the extent to which young infants remember what they learned days before. When the opportunity is given to encode more cues during training, young infants’ long-term memory is expanded, suggesting that the forgetting of young infants is not an availability issue, but an accessibility failure. In summary, infant memory is not an unformed version of human development but is recognized as a critical aspect of development with functional similarities to adult memory.

## Time Windows

**Figure 2:**
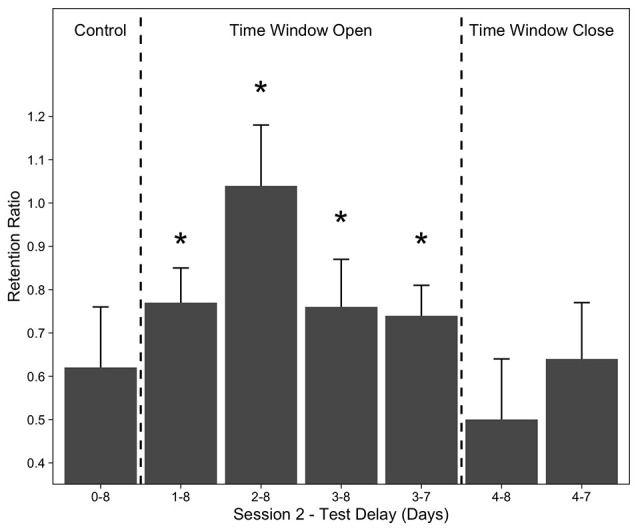
Mean retention ratios of 3-month-olds whose second training session followed their first by either 1, 2, 3, or 4 days and control group who received no second training session. Asterisks indicate that groups whose second session occurred within 3 days of Session 1 exhibited significant retention. Error bars represent ±1 SE. Redrawn from Rovee-Collier (1995). Copyright (1995) Elsevier.

Rovee-Collier introduced the concept of time windows to identify the critical periods when different events or pieces of knowledge are integrated (for a review see Rovee-Collier, 1995). After encountering a piece of information, if a new encounter occurs outside the critical time window (e.g., 4 days after the initial encounter at 3 months of age, Rovee-Collier et al., 1993a), these two instances are perceived as two separate events or representations. If the new encounter occurs within 4 days following the first encounter, then these two events are integrated and formed one memory representation. The integration of two temporally distinct events may have a wide range of outcomes, such as learning (Rovee-Collier et al., 1995), forming and expanding category representations (Rovee-Collier et al., 1993a), memory modification (Rovee-Collier et al., 1994; Boller et al., 1995; Muzzio and Rovee-Collier, 1996) and retrieval success (Rovee-Collier et al., 1980; Greco et al., 1986). An important example of the role of learning and memory is that, if the time interval between the first and second training session was less than 3 days, these two event representations were integrated and the training mobile was remembered for the next 8 days (Rovee-Collier et al., 1995). On the fourth day, however, the time window closed and the infant did not remember the training mobile at the 8-day retention test (Figure 2).

Modification of prior memories is another function of the time windows. For instance, when adults are presented with conflicting or misleading information after an event, their memory of the previous event could change drastically (Loftus, 1975, 1979). Being exposed to post-event information could also change the memories of young infants if the exposure is within a certain time window. After training with the same mobile for consecutive days, infants who encountered the novel mobile after less than 3 days remembered both the training and novel mobiles in the future (Rovee-Collier et al., 1994). A more than the 3-day interval between training and post-event information (novel mobile) resulted in disruption of the training memory. Infants only remembered the mobile presented as post-event information. At 6 months of age, when the post-event information (novel mobile) was presented immediately after training, infants still remembered both memories of the training and novel mobile (Boller et al., 1995). However, after intervals of more than a day, the infants did not remember the training mobile anymore (Muzzio and Rovee-Collier, 1996). Taken together, these studies support the idea that memories of young infants are also integrated with novel information in a way that both representations are intact only if the post-event information is encountered within a specific time window. Outside of this time window, only recent events are emphasized, at the cost of prior experiences. Infants could update their behavior when the memory of prior experiences becomes fuzzy with time and, in that case, new sensory information is weighted more heavily in the final memory representation.

The evidence presented in this section suggests that the time window is an important cognitive construct in early development and indicates when infants integrate separate events or representations to form category representations, to enhance long-term memory, and to be reminded of previous encounters. According to Rovee-Collier (1995, p. 166) time windows are the “mortar that holds together the separate building blocks of cognitive development.” In these critical periods, subsequent encounters could not only facilitate the memory of an event but also alter it. Given that the focus of these findings is a group-level analysis of the behavior of young infants, what is now needed is an investigation of individual differences in time windows and how these differences are important for later cognitive functions (e.g., intelligence and working memory). Overall, forming memories of past events is a time-sensitive process, and frequent exposures to the elements of the original event support long-term memory consolidation during early infancy.

## Generalization and Visual Context

Young infants encode not only the proximal cues, such as the mobile itself, but also distal cues, such as the bumper in the crib and the room in which the learning occurs to function as effective reminders in the future (Rovee-Collier et al., 1985; Rovee-Collier and Hayne, 1987; Hayne et al., 1991). For example, when they encountered the same crib bumper as in the training, this familiar cue helped them remember the training mobile (Fagen et al., 1976; Hayne et al., 1986). In addition to the visual features of the environment, correlated attributes of these features (e.g., red block always presented with “+” figure on the block) are learned by young infants (Bhatt and Rovee-Collier, 1994, 1996, 1997). When these features are highly distinctive (e.g., unfamiliar and colorful liner in the crib presented to infants), they showed significant retention for the specific details of the learning episode at 3 months (Butler and Rovee-Collier, 1989) and 6 months of age (Boller et al., 1990). Similarly, distinctive local features on a block of the mobile among distractor blocks (e.g., Q figure among O figures and R figure among P figures) created a pop-out effect, resulting in better retention due to in-depth processing (Adler et al., 1998a,b; Gerhardstein et al., 1998, 1999; Rovee-Collier et al., 1999). Furthermore, both the auditory context created by playing the same musical piece (Fagen et al., 1997) and the olfactory context in which infants smelled the same ambient odor (Rubin et al., 1998; Schroers et al., 2007; Suss et al., 2012) helped infants remember the mobile that they encountered days before if these contexts were shared between the training and test sessions. As a result, retention in early infancy is significantly facilitated by the availability of cues only if the test session consisted of the cues from training. These results are in line with the argument that an event is more likely to be remembered when the learning and retrieval episodes are highly similar, which refers to the principle of encoding specificity introduced in adult memory research by Tulving (1972). Moreover, a specific visual characteristic of the environment such as distinctiveness of the visual context (e.g., linen draped over the crib) and stimuli (e.g., pop-out block in the mobile) could enhance the retention.

The visual cues that are incorporated into memories can help infants generalize their response to another mobile encountered in the future (Fagen et al., 1984; Hayne et al., 1986). For example, 3-month-old infants responded at the same rate of kicking as in the training when they were tested with the novel mobile 4 days after the training ended only when the general features of the memory remained and the specific features were forgotten (Rovee-Collier and Sullivan, 1980). Another similar generalization effect has been observed when infants are presented with multiple different mobiles during training (Fagen et al., 1984; Hayne et al., 1987; Greco et al., 1990; Rovee-Collier and Dufault, 1991; Rovee-Collier et al., 1993a, b; Merriman et al., 1997). In a more recent study, 3–4-month-old infants generalized their kicking response to a test mobile when the toys faced the infant from an angle different than the wide range of viewpoints available in the training mobile (Kraebel et al., 2007).

There are also cases in which the memory of the training and cues in the test session differ so that young infants’ response level returns to baseline, suggesting that they distinguished subsequent cues from the memory of the training. The visual cues facilitated the novelty detection when a novel mobile was presented after the training with the same mobile over successive days (Rovee and Fagen, 1976; Rovee-Collier and Sullivan, 1980; Fagen et al., 1984), the number of toys in the mobile decreased from training to test (Mast et al., 1980), and local features (color and shape) of the linen draped over the crib was different during the long-term retention test (Rovee-Collier et al., 1992). Thus, infants distinguish an encounter that differs from what they learned in the past because their memories also help them create expectations for future events (Mast et al., 1980; Fagen et al., 1984; Fagen, 1993). Violation of these expectations (e.g., number of toys decreased from 10 to 2) resulted in crying and fussiness (Fagen and Ohr, 1985; Singer and Fagen, 1992). This negative reactivity also disrupted the retention for the learned mobile a week later (Fagen et al., 1985, 1989).

Infants’ ability to learn and retain memories along with the information about where an event took place (e.g., visual context information, a place where the experiment is held) contradicts the ideas of the time claiming that the retention capacity for place information is limited in the first year of life due to the immaturity of the hippocampus (Nadel and Zola-Morgan, 1984). The argument that the retention capacity for declarative memories develops towards the end of the first year of life was supported recently (Bauer, 2006, 2008), and it was still considered the dominant view concerning infant memory (Mullally and Maguire, 2014). Recent arguments against the late maturation of the memory system have been provided by Hayne (2004). Variables that influence the declarative memory performance of adults, such as age, length of the retention interval, and whether the context changed from training to test, have been argued to also affect the memory performance of young infants in the mobile paradigm, suggesting that higher-order memory skills that require conscious recollection as in declarative memory exist in young infants. The lack of consensus in this long debate highlights that targeted research on the development of explicit memory in early infancy is needed to resolve the issue.

All of the studies reviewed in this section suggested that the memories of young infants consist of global and local visual cues of their immediate surroundings. Depending on the extent to which the cues of the environment and memory are compatible, infants either detect the distinctive features or generalize their response, and violation of their expectations of the consequences of the task may result in negative emotionality.

## Summary of Rovee-Collier’S Work on Mobile Paradigm

When Rovee-Collier started her systematic investigation of infant memory, young infants’ capacity for encoding and retrieving information was considered to be quite limited (Nadel and Zola-Morgan, 1984; Schacter and Moscovitch, 1984). In contrast to these ideas, research on infant memory conducted by Rovee-Collier and colleagues argued that infants have a capacity for learning visual cues and the context surrounding them (Rovee-Collier et al., 1985; Hayne et al., 1986; Butler and Rovee-Collier, 1989; Hayne and Rovee-Collier, 1995; Hartshorn et al., 1998b). The infants gradually forget what they learned (Rovee-Collier and Sullivan, 1980; Rovee-Collier et al., 1981; Boller et al., 1990), but remember for a long time when they are reminded with effective cues (Rovee-Collier et al., 1980; Fagen and Rovee-Collier, 1983; Galluccio and Rovee-Collier, 1999), and their memories are modified by encountering novel information (Rovee-Collier et al., 1993a, 1994; Boller et al., 1996; Muzzio and Rovee-Collier, 1996). Although some developmental differences have been observed concerning the temporal parameters of these basic memory processes (Fagen and Rovee-Collier, 1983; Boller et al., 1990), Rovee-Collier and colleagues claim that the same principles apply to the memory systems of both infants and adults (Rovee-Collier, 1997; Rovee-Collier and Hartshorn, 1999; Rovee-Collier and Barr, 2001). These findings are in line with the idea that pre-verbal infants’ memory capacity is more than just habit or motor learning (Meltzoff, 1985; Nelson, 1995; Thelen, 2000; Hellmer et al., 2018). They are also in parallel to the results of other declarative memory measures (e.g., imitation task) that were used to study the memory capacity of pre-verbal infants (Meltzoff, 1985; Bauer and Mandler, 1989). For instance, studies with both imitation tasks (Bauer, 2015) and mobile tasks (Boller et al., 1990) showed that how long the memory will be remembered increases with age. Likewise, the serial order of the events/actions are learned in both of these tasks (Gulya et al., 1998; Carver, 1999).

## Recent Years

The recent literature which grew outside of the work by Rovee-Collier and colleagues has taken the sensorimotor experience into the center by investigating how the ability to learn sensorimotor contingencies can answer questions about motor and cognitive development at large. This section first gives a brief overview of recent research that has focused on mechanisms underlying learning sensorimotor contingencies and their possible cognitive outcomes. We then review the literature on motor development, more specifically research that used the mobile paradigm as a way of understanding the ontogeny of motor behavior.

Emerging findings shed light on possible learning mechanisms that may be at work while learning sensorimotor contingencies in the mobile paradigm (Kelso and Fuchs, 2016; Kelso, 2016; Zaadnoordijk et al., 2018). Some findings based on computer simulations show that increased movement in the contingent phase could be explained by reinforcement learning that does not require any representation of the cause and effect relationship (Zaadnoordijk et al., 2018). Earlier accounts of learning in the mobile paradigm were in line with this argument. Operant conditioning was considered the underlying learning mechanism, and movement of the mobile was considered to function as the reinforcer, leading to the gradual increase in the movement rate (Rovee and Rovee, 1969; Fagen et al., 1976; Hayne, 2004). Both gaining control over one’s actions (Rovee-Collier and Gekoski, 1979) and the context constituting the visual aspects of the infant’s environment (Rovee-Collier et al., 1985) contributed to the reinforcement value of the moving mobile. This interpretation differs from that of Kelso (2016), who asserted that, in the mobile paradigm, the infants realize that they can change the environment with their own movements and, therefore, can infer causality from the relationship between the actions and their consequences. Empirical investigations into the role of causal learning on the mobile paradigm have examined whether infants’ expectations are violated when the movement of the mobile is not contingent upon their actions (Zaadnoordijk et al., 2020). When the mobile was disconnected, electroencephalography showed mismatch negativity pointing to violation of expectation, along with a movement burst in the connected limb, suggesting that the infant’s predictions of the cause and effect model were violated. Despite being few and without consensus, these findings provide important insights into the role of learning sensorimotor contingencies in the mobile paradigm on infant cognitive development. Further research could benefit from modeling empirical data to examine how these learning mechanisms differentially affect detecting and remembering the contingent relationship between infants’ actions and their consequences.

To date, several studies have examined the kinematics of movement in the mobile paradigm to shed light on how motor development is affected by the infants’ interaction with the environment (Watanabe and Taga, 2006, 2009, 2011; Watanabe et al., 2011). Some other researchers were specifically interested in the movement patterns when the mobile paradigm had specific task requirements. In these experimental designs, only the movements that satisfied particular criteria, such as the angle between the upper and lower leg exceeding a threshold (Thelen, 1994; Angulo-Kinzler and Horn, 2001; Angulo-Kinzler, 2001; Angulo-Kinzler et al., 2002; Tiernan and Angulo-Barroso, 2008), keeping the foot (Sargent et al., 2014, 2015) or head (Tripathi et al., 2019) above a virtual threshold, and foot contact with a touch panel (Chen et al., 2002), were able to activate the mobile. For example, Angulo-Kinzler (2001) developed the constrained version of the mobile paradigm in which the legs of the infants activated the mobile only if the extension or flexion of the leg exceeded a particular angle threshold. Though some infants executed small movements around the threshold without increasing the overall movement rate, others preferred large extensions and flexions that resulted in increased movement frequency (Angulo-Kinzler and Horn, 2001; Angulo-Kinzler et al., 2002). As a result, infants not only increased the movement frequency while learning the contingency in the mobile paradigm but also developed unique and adaptive motor solutions according to the task requirements, suggesting a role of individual differences in learning sensorimotor contingencies.

Recent technological developments enabling more sensitive measurements have led to a renewed interest in individual differences in the learning and retention abilities of infants in the mobile paradigm. Given that previous reports have indicated that not all infants can learn the contingency in the mobile paradigm (Gerhardstein et al., 2012; Jacquey et al., 2020a), investigating behavioral differences between learners and non-learners has gained importance. In a study in which the mobile was activated only if the target leg exceeded a virtual threshold, infants who were able to learn the task exhibited different movement kinematics (e.g., less in-phase hip-knee coordination) compared to non-learners during the acquisition phase (Sargent et al., 2015). In another study in which the infants were required to move their heads above a virtual threshold, 50% of them did not satisfy the learning criteria (Tripathi et al., 2019). During the acquisition phase, learners increased the amount of time that they reactivated the mobile by exceeding the virtual threshold with their heads, whereas non-learners maintained their baseline level. Similarly, Watanabe and Taga (2011) demonstrated that infants whose average arm movements had high velocity during baseline did not increase their movement rate in the acquisition phase, whereas infants with low-velocity arm movements increased their overall movement rate, suggesting that they were able to detect the causal relationship between their arm movements and the movement of the mobile. Overall, individual differences in the movement characteristics of the infants (e.g., velocity of the limbs) are related to whether infants can learn the contingency between their actions and consequences.

How these individual differences predict later motor and cognitive development has also received some attention. For example, a longitudinal study demonstrated that immediate retention capacity in the mobile paradigm is related to motor development, verbal skills, and intelligence at 24 and 32 months of age (Domsch et al., 2009). Similarly, the amount of reaching increased at 3 months of age when infants were trained with a mobile contingent upon their arm movement, suggesting that sensorimotor learning could facilitate the development of other motor behaviors (Needham et al., 2014). The intriguing question of how learning and remembering sensorimotor contingencies affect future behavior can be explored usefully in further research, especially when the limited number of longitudinal studies on this topic is taken into consideration.

Using the mobile paradigm, researchers have also been able to investigate how different age groups and populations differ in terms of their motor behavior while learning sensorimotor contingencies. Watanabe and Taga (2006) argued that, from 2–4 months of age, the motor behavior of young infants follows a general to a specific trend. More specifically, 2-month-old infants increased the movement frequency of all limbs during the acquisition phase, and movement increased in the specific arm connected to the mobile only at 4 months. Similarly, limb differentiation was observed in infants aged 4–8 months in a contingency detection task in which the presentation of an audio-visual stimulus was contingent on the movement of a particular arm (Jacquey et al., 2020b). Despite the failed attempt of Jacquey et al. (2020b) to replicate the developmental trend observed in Watanabe and Taga (2006), both studies showed that the limb differentiation started at 4 months of age. Previous results reported by Rovee-Collier et al. (1978) showed that such limb differentiation is evident even at 3 months. The most obvious conclusion to emerge from these studies is that learning in the mobile paradigm is not the result of a joy reaction (Piaget, 1952) because infants can increase the movement of a particular limb while keeping the others at the baseline level. However, it is also important to note that age-related differences concerning such limb differentiation are inconclusive, and one likely explanation may be that the methodological parameters varied among studies. For example, Watanabe and Taga (2006) used a motion tracking system to analyze the movement of infants, and Jacquey et al. (2020b) measured the arm activity with accelerometers, whereas Rovee-Collier et al. (1978) relied on more traditional methods, such as counting the number of kicks. These findings indicate a need for systematic replications with modern techniques to gain a more nuanced understanding of motor learning across different age groups.

## Critique on Mobile Paradigm Studies

The most comprehensive criticism of the mobile paradigm is that unlike memory tasks (e.g., imitation task), it does not measure declarative memory (Bauer et al., 2007). This critique of Rovee-Collier and colleagues has endured from the early years of the mobile paradigm. Neuro-maturation accounts have argued that explicit/declarative memory is not yet fully formed during early infancy (Schacter and Moscovitch, 1984) and that the mobile paradigm only measures procedural/habitual memory Bauer (1996, 2004, 2007) and Bauer (2008) further argues that learning and memory measured with kicking behavior do not have any representational components and that infants’ actions in the mobile paradigm might rely on brain structures, such as the cerebellum, which mature quite rapidly in the early stages of development.

Other criticism has focused more on methodological challenges. The mobile paradigm relies on cross-sectional data targeting specific age groups and is limited to group-level analysis. As a result, individual differences in learning and memory performance of infants are mostly ignored. For example, a meta-analysis conducted with mobile paradigm studies showed that 15% of the infants were excluded from the analysis because they did not learn the task (Gerhardstein et al., 2012). In some cases, the infants who did not satisfy the learning criteria constituted 25% (Gerhardstein et al., 1998; Hildreth et al., 2003), 30% (Gulya et al., 1998; Hildreth et al., 2003; Cuevas et al., 2015), 44% (Sweeney and Rovee-Collier, 2001) and even 50% of the sample (Tripathi et al., 2019). According to the learning criteria, infants were expected to exceed 1.5 times their baseline movement rate at any two consecutive minutes of the acquisition phase (Rovee-Collier et al., 1985; Hayne et al., 1986). This definition focuses on operant conditioning where the movement of the mobile functions as the reinforcer (Rovee and Rovee, 1969; Fagen et al., 1976). However, this approach can only draw a very limited picture of learning and memory in the mobile paradigm for two reasons. First, it discounts situations where infants use different learning strategies, especially the ones that can cause nonlinear behavior. Second, if the sample consists of only infants who can learn the task, in other words, the ones who satisfy the learning criteria, then the conclusions are limited to the behavior of those who were able to satisfy the criteria for operant conditioning. Thus, it remains unknown to what extent these findings are generalizable and what infants actually know and can learn.

A recent critique of the replicability of this learning effect by Jacquey et al. (2020a) pointed to the publication failures of several research groups due to unreliable learning effects. They noted that learning and memory of infants in the mobile paradigm were inferred from observational techniques (e.g., counting the number of kicks), with an imprecise operational definition of kicking behavior. For instance, recent pilot work in our lab with the traditional mobile setup showed that infants performed actions different than kicking (e.g., small foot movements when the leg is in an extended position) to activate the mobile. Second, control groups were presented with the stimulus manually, in a way that the experimenter moves the mobile at a particular rate (Rovee and Rovee, 1969; McKirdy and Rovee, 1978; Rovee-Collier et al., 1978) which might have resulted in unintended biases in implementing the experimental procedure. These control studies were the first ones testing and supporting the claim that infant learning during the mobile paradigm was a result of the contingency rather than caused by other factors such as excitement, visual or haptic stimulation. However, this assumption has not been tested with technological tools that allow in-depth analysis of movement characteristics and controlling for other variables that could lead to movement increase such as haptic feedback on the limbs.

The two most commonly used memory measures in the mobile paradigm (baseline and retention ratio) have been criticized for overestimating the strength of the retention (Bogartz, 1996) which put the reliability of these measures into question. Bogartz (1996) argued that even when memory strength is zero (e.g., when forgetting is complete), it is mathematically implausible for the retention ratio to be zero because the value (e.g., kicking rate) in long term retention test divided by another value in immediate retention test will be more than zero. Moreover, retention tests that are introduced right after the learning takes place (e.g., immediate retention test) usually facilitate memory consolidation and the strength of the memory. Retention ratio relying on the outcome of immediate retention test would be contaminated by this consolidation process, thus overestimating the memory strength.

It is also worth noting that the literature on the mobile paradigm has often involved Rovee-Collier and her collaborators both during her time and after her death in 2014 (Vitello, 2014). Replications from within the same research group are more likely to result in replication success than from other research groups because researchers within Rovee-Collier’s group might be more hesitant about publishing results that are contradictory to their previous findings (Ioannidis, 2005; Makel et al., 2012). Thus, there is a need for both exact and conceptual replications by other scientific teams. Furthermore, replication of the key findings in the mobile paradigm literature with new behavioral, neural, and computational methods and advanced analysis techniques seems to be crucial for evaluating previous findings and developing new protocols.

In addition to methodological issues, we would like to bring up an additional point of critique that is not raised in the literature so far. The sensorimotor aspect of the mobile paradigm is mostly neglected and results have often been interpreted as a sign of perceptual and memory processes of young infants (Rovee-Collier and Sullivan, 1980; Rovee-Collier et al., 1981; Fagen and Rovee-Collier, 1983; Suss et al., 2012; Merz et al., 2017; Tripathi et al., 2019). Motor behavior was even considered a substitute for verbal behavior. Rovee-Collier (1997, p. 471) stated that “Infants “tell” us whether or not they recognize the test mobile […]. If infants recognize the test mobile, then they say “yes” by kicking at a rate higher than their individual baseline rates; if they do not recognize the test mobile, then they say “no” by not kicking above their baseline rates.” The fact that the mobile paradigm was not associated with its sensorimotor nature is paradoxical not only because sensorimotor learning is the backbone of the paradigm, but also because the outcome variable is a kinematic measure (e.g., number of kicks). The key problem with this view is that it creates a gap between knowledge representations acquired through high order cognitive processes (e.g., perceptual learning, declarative memory, category learning), sensorimotor experience, sensorimotor memories, and procedural learning. Furthermore, it overlooks the importance of the infant’s dynamic relationship with the world in the early stages of development (Piaget, 1952; Thelen et al., 2001; Thelen, 2005). The lack of emphasis on sensorimotor experience in the work by Rovee-Collier and colleagues might be considered the product of the research zeitgeist of the time. It is in line with classical accounts of cognition which assert that motor behavior is only a medium to form amodal knowledge representations (Fodor, 1983).

## New Embodied Account on Mobile Paradigm

The last two decades have seen a growing trend towards embodied accounts of cognition and development. It has been argued that knowledge representations are modal and dependent on action and perception (Niedenthal et al., 2005; Barsalou, 2010). Moreover, the role of motor experience (Von Hofsten, 2004; Sommerville et al., 2005; van Elk et al., 2008) and embodiment (Thelen et al., 2001; Smith, 2005; Westermann et al., 2007; Gredebäck and Falck-Ytter, 2015; Gottwald et al., 2016; Corbetta et al., 2018) on cognitive development has received a lot of attention. Together, these perspectives claim that the body is not only a tool to understand the higher-order cognitive processes but it is a crucial aspect of the infant’s dynamic sensory interaction with the world. We propose a new embodied account of the mobile paradigm which shifts the spotlight from classical accounts of cognition to an understanding that puts the infant’s active exploration and ability to detect sensorimotor contingencies at the center. This new account aims to take the mobile paradigm out of its confined and dis-embodied context and place it in a broader one, which takes sensorimotor experience and variability into consideration while arguing for new mechanistic explanations.

The new embodied account draws support from other studies in the field of infancy research arguing that the contingent relationship between body and environment forms the basis of learning in the very first months of life. When newborn infants direct their arm movements towards a light source this is done to facilitate the coupling between proprioceptive feedback and visual information (van der Meer et al., 1995; van der Meer, 1997). This visuomotor coupling produced by contingencies between self-initiated movements and environmental feedback has been proposed to constitute the emergence of reaching (Corbetta et al., 2014). Similarly, when learning the correspondences between the body and immediate environment, facilitates the development of body representations (Thomas et al., 2015; Hoffmann et al., 2017). While arm-based learning is more prominent at the beginning of life (Rochat, 1993), caudal body parts (e.g., hips, legs) are integrated into infants’ body representation as infants gain more experience with leg-based learning (Watanabe and Taga, 2011; Thomas et al., 2015). Recently, studies focusing on the ability to learn contingencies revealed that detecting the contingency between a specific limb movement and mobile is an ability that develops with age (Watanabe and Taga, 2006; Jacquey et al., 2020b). Development involves an ever-changing process of interaction between perception and action. Measuring exactly the capacity for such integration, the mobile paradigm offers an opportunity to understand how learning sensorimotor contingencies unfold in real-time.

Pioneering work on the dynamic systems theory of development has made it evident that interaction of multiple factors (e.g., physical characteristics of infant, environment) and variability are crucial aspects for the emergence of new behavior (Thelen, 1995; Smith and Thelen, 2003). For instance, submerging an infant’s legs in the water results in more stepping behavior whereas increasing the weight of the body leads to less kicking at 6 weeks of age, suggesting that even a minor change could impact the system considerably (Thelen et al., 2002). Furthermore, the variability in performed actions increases before a particular motor behavior is learned (Thelen, 1979). The work done by Rovee-Collier and colleagues never goes beyond the group-level analysis while investigating the learning and memory processes of young infants. Contrary to this, we argue that the variance and multiple factors that affect the emergence of new motor behavior are keys to unfolding the mobile paradigm and examining how young infants explore themselves and the world around them. Some studies in recent years have initiated the process of closing this gap in the literature. For example, it was emphasized that individual differences can be observed in the motor preferences of young infants while learning contingencies in the mobile paradigm (Thelen, 1994; Angulo-Kinzler and Horn, 2001; Watanabe and Taga, 2011).

It has been established that the visual system has two functionally distinct pathways in the cerebral cortex: the ventral pathway is for visual recognition and the dorsal pathway is for guiding action such as reaching for an object (Milner and Goodale, 1993, 2008). More recently, it is also acknowledged that these two streams continuously interact (Adamo and Ferber, 2009; Kitadono and Humphreys, 2009). The dorsal stream uses visual information for motor planning, guiding the body in space while executing motor behavior. Similarly, while infants in the mobile paradigm learn contingencies following visual and haptic feedback, they learn about their own bodies and affordances of their actions at the same time. Like learning any type of action, learned behavior in the mobile paradigm is retained over time and can be remembered in the presence of visual cues such as the room where the experiment took place or the linen draped over their cribs (Fagen and Rovee-Collier, 1983; Hayne et al., 1986; Butler and Rovee-Collier, 1989). The representation of learned actions decays over time (Rovee-Collier et al., 1981; Boller et al., 1990) and can be reestablished after a few trials when infants are reminded of learning context (Rovee-Collier et al., 1980; Fagen and Rovee-Collier, 1983; Muzzio and Rovee-Collier, 1996). Thus, both visual recognition capacity and guiding the action following the proprioceptive and visual feedback are important aspects of the mobile paradigm. We argue here that the dorsal stream may play a central role in learning sensorimotor contingencies in the mobile paradigm due to the similarity between functional properties of the dorsal stream and behavioral components of the mobile paradigm.

To sum up, we suggest an alternative view of the mobile paradigm which can be used for a more comprehensive analysis of motor behavior and related behavioral consequences. Instead of relying on the classical understanding of cognition, where actions are used to infer abstract and dis-embodied forms of symbolic cognition, we argue instead that sensorimotor experience, actions, and their variability should define the functional characteristics of the paradigm. Furthermore, we argue that this new description likely employs mechanisms that are responsible for guiding the action and body in space, as per the visual input. Recent literature both in developmental science in general and the mobile paradigm, in particular, is in line with this new embodied account and can be considered as an inevitable backlash against the traditional views which had overlooked the role of embodied experience in learning and memory. Concerning the interpretation of infant’s behavior in the mobile paradigm, we provide a different interpretation than Rovee-Collier. We suggest that what has been measured all along is the ability to learn to act and interact, the development of a dorsally driven action memory, not the ability to represent and remember an abstract, adult-like manner.

## Conclusions

Half a century ago, young infants’ performance in the mobile paradigm started to challenge the accounts underestimating the learning and retention capabilities of infants in the first months of life. Recent findings using the mobile paradigm have opened up new discussions enabling in-depth analysis of the development of motor and cognitive skills through early infancy, more specifically by focusing on individual differences in learning sensorimotor contingencies. This review attempts not only to highlight the importance of the mobile paradigm in understanding cognitive and motor development of young infants but also to expose methodological and theoretical limitations of the literature which disregard the role of action and embodiment. Here, we proposed a new embodied account on the mobile paradigm that shifts the focus from what external behavior could reveal about the cognitive processes to an understanding of the development by incorporating action, representation, and variance.

## Author Contributions

US was responsible for writing the original draft, editing the manuscript, and visualization of graphs. GG was responsible for conceptualization, reviewing, and editing the manuscript. All authors contributed to the article and approved the submitted version.

## Conflict of Interest

The authors declare that the research was conducted in the absence of any commercial or financial relationships that could be construed as a potential conflict of interest.
