# Corrigendum: Making the world behave: A new embodied account on mobile paradigm

**DOI:** 10.3389/fnsys.2023.1134410

**Published:** 2023-02-21

**Authors:** Umay Sen, Gustaf Gredebäck

**Affiliations:** Department of Psychology, Uppsala University, Uppsala, Sweden

**Keywords:** mobile paradigm, sensorimotor contingency, embodiment, infant memory, learning

In the published article, there was a typographic error. The word “phone” was mistakenly inserted after “mobile”. A correction has been made to the section **The Mobile Paradigm**, paragraph 2. The corrected paragraph is below.

“In practice, the procedure is as follows. After the infant is placed in a crib, a ribbon is attached to one of their legs. Two adjacent stands are mounted on the crib, one connected to a mobile, and the other is empty. The original (and most used) paradigm has three phases: baseline (3 min), acquisition (9 min), and extinction (3 min; Fagen et al., 1976; Sullivan et al., 1979). During the baseline and extinction phases, the infants are allowed to move their legs normally with their legs attached to the empty stand. During the acquisition phase, the leg is connected to the mobile stand and their movements set the mobile in motion. Rovee-Collier argued that, after operant learning took place (e.g., increased kicking rate in the first minutes of acquisition phase) by gaining control over the mobile, the environment continued to reward the infant, resulting in individual differences in movement with respect to how much the infant experimented with their surroundings. Not only the sensory consequences (e.g., haptic feedback in the leg, visual stimulation coming from the moving mobile), but “making the world behave” (Skinner, 1953, as cited in Rovee-Collier and Gekoski, 1979), in other words gaining control over the mobile, strengthened the stimulus-response associations.”

The authors apologize for this error and state that this does not change the scientific conclusions of the article in any way. The original article has been updated.

